# Physical activity and health-related quality of life among high-risk women for type 2 diabetes in the early years after pregnancy

**DOI:** 10.1186/s12905-022-01664-7

**Published:** 2022-03-21

**Authors:** Niina Sahrakorpi, Elina Engberg, Beata Stach-Lempinen, Tuija H. Tammelin, Janne Kulmala, Risto P. Roine, Saila B. Koivusalo

**Affiliations:** 1 Helsinki University Hospital, Helsinki, Finland; 2 City of Vantaa Preventive Medical Services, Communal Maternity Care, Kielotie 40, 01300 Vantaa, Finland; 3 Folkhälsan Research Center, Helsinki, Finland; 4 Department of Psychology and Logopedics, University of Helsinki, Helsinki, Finland; 5 Department of Obstetrics and Gynecology, South Karelia Central Hospital, Lappeenranta, Finland; 6 JAMK University of Applied Sciences, LIKES, Jyväskylä, Finland; 7 Department of Health and Social Management, University of Eastern Finland, Kuopio, Finland; 8 Department of Obstetrics and Gynecology, University of Helsinki, Helsinki, Finland

**Keywords:** Obesity, Women, Gestational diabetes, HRQoL, Postpartum, Sedentary behaviour, 15D, Device-measured physical activity

## Abstract

**Background:**

Previous studies have shown that physical activity (PA) correlates positively with health-related quality of life (HRQoL) in the general population. Few studies have investigated associations between device-measured PA and HRQoL among premenopausal women at risk for type 2 diabetes (T2D). In addition to physical well-being, general well-being improved by PA has been suggested to strengthen PA’s benefits in reducing metabolic diseases. The aim of this study was to examine the associations between PA and HRQoL (general and dimensions) among high-risk women in the early post-pregnancy years when T2D risk is highest and to estimate whether current obesity or prior gestational diabetes (GDM) modified these associations.

**Methods:**

This cross-sectional study of high-risk women [body mass index (BMI) ≥ 30 kg/m^2^ and/or prior GDM)]4–6 years after delivery measured sleep, sedentary time, daily steps, and light (LPA), moderate-to-vigorous (MVPA), and vigorous PA (VPA) with the SenseWear ArmbandTM accelerometer for seven days and HRQoL with the 15D instrument.

**Results:**

The analyses included 204 women with a median (IQR) age of 39 (6.0) years and a median BMI of 31.1 kg/m^2^ (10.9). 54% were currently obese (BMI ≥ 30 kg/m^2^), and 70% had prior gestational diabetes (GDM+). Women with obesity had lower PA levels than women with normal weight or overweight (*p* < 0.001) but there was no difference between the GDM+ or GDM− women. Women with both current obesity and GDM+ had highest sedentary time and lowest PA levels. The whole sample’s median 15D score was 0.934 (IQR 0.092), lower among women with obesity compared to the others (*p* < 0.001), but not different between GDM+ or GDM−. There was a positive correlation between VPA (adjusted r_s_ = 0.262 *p* = 0.001) and the 15D score. After grouping according to BMI (< and ≥ 30 kg/m^2^), the associations remained significant only in women without obesity. Among them, sleep, total steps, MVPA, and VPA were positively associated with 15D.

**Conclusions:**

Higher PA levels are associated with better HRQoL among high-risk women with normal weight and overweight but no differences were found among women affected by obesity in the early years after pregnancy.

*Trial registration* Ethics committees of Helsinki University Hospital (Dnro 300/e9/06) and South Karelian Central Hospital (Dnro 06/08).

## Introduction

Along with the obesity pandemic, the incidence of type 2 diabetes (T2D) has increased globally [1, 2], burdening both the health care system and those suffering with the disease [1, 2]. T2D is a heterogenous disorder [3–5], and in addition to genetic factors [1, 2], health behaviours such as low level of physical activity [1, 2], and unhealthy diet [1, 2] contribute to the onset of T2D.

According to previous systematic reviews, moderate-to-vigorous physical activity (MVPA) and vigorous PA (VPA) are associated with a 25–40% reduction in the risk of T2D in the general population [6, 7]. The benefits of PA have also been demonstrated on health-related quality of life (HRQoL) [8–10], regardless of weight status [11], and in older, high-T2D-risk populations [12]. Most previous studies of the relationship between PA and HRQoL have used self-reported PA measures [8–12]. A direct inverse association between T2D risk score and HRQoL has been detected, and lack of PA, obesity, and a history of high blood glucose were the factors most clearly associated with lower quality of life in the general population [13]. Thus, it has been suggested that improved subjective well-being gained by PA may reinforce the physiological benefits of PA on risk reduction for later metabolic disturbances, including T2D [11, 12, 14].

The prevalence of maternal obesity and gestational diabetes (GDM) has increased along with the T2D epidemic [1]. Maternal obesity and GDM are independent and well-known risk factors for later T2D, and the risk is highest in the early years after pregnancy [1, 2, 15]. According to a recent meta-analysis by Vounzoulaki et al., women with a history of GDM had a 17-fold risk of developing further T2D within 5 years after pregnancy [16], highlighting the importance to prevent T2D among these women especially in the early years after pregnancy. Although lifestyle choices, including diet and PA, have a substantial role in the risk of eventual progression of impaired glucose tolerance to T2D [1, 2], and women with obesity and gestational diabetes are likely motivated to receive lifestyle guidance during pregnancy, only a small proportion of these women follow the recommendations after delivery. According to an Australian survey, 6–24 months after having been treated for GDM, only approximately 1 out of 3 women reported sufficient PA levels [17]. Caregiving duties and lack of assistance with children, energy, and time are common reasons for inactivity among women with young children [12, 18]. From previous studies we know that women with obesity are at risk for declining HRQoL [19, 20]. When planning interventions to postpone or prevent T2D, it is essential to know whether PA increases the subjective well-being among the high-risk women, which could motivate them to engage in active lifestyle during these busy but important first postpartum years. However, there is a lack of studies examining this relationship between PA and HRQoL among high-risk women in early years after pregnancy.

Therefore, the aims of this study were (1) to investigate the associations between different intensities of device-measured PA and HRQoL (general and dimensions) in high T2D risk women four to six years after pregnancy, and (2) to study whether obesity or prior GDM modified these associations.

## Materials and methods

This cross-sectional study utilized postpartum follow-up data 4–6 years postpartum from the Finnish Gestational Diabetes Prevention Study (RADIEL), conducted between 2008 and 2014 at Helsinki University Hospital and South Karelian Central Hospital. The main focus of RADIEL was to study the effectiveness of early lifestyle interventions in the prevention of GDM. Subjects in the intervention group received individualized counseling on diet, PA and weight control from trained nurses, and the control group received standard antenatal care The details of the original RADIEL study have been published previously [21].

### Participants

Altogether 720 women were recruited to the original study. The inclusion criteria were over 18 years of age, at high risk of diabetes (BMI ≥ 30 kg/m^2^ and/or prior GDM), either planning a pregnancy (*N* = 228) or in early pregnancy before 20 gestational weeks (*N* = 492). The original study’s exclusion criteria were current diabetes, medication altering glucose metabolism, multiple pregnancy, severe psychiatric state, physical disability, and significant difficulty in co-operation (e.g., inadequate Finnish language skills).

Between 2013 and 2017, we invited the 596 RADIEL participants with a live birth to a follow-up study 4–6 years after their delivery. Both intervention and control groups received invitation to the follow-up. Altogether 348 (58.4%) participants responded to the invitation. Several indicators were used to assess metabolic, cardiovascular, mental, and lifestyle outcomes at the follow-up. This analysis is based on the follow-up data and comprises women who had valid device-measured PA and HRQoL data.

All subjects gave written informed consent. The study complied with the Declaration of Helsinki and received approval from the ethics committees of Helsinki University Hospital (Dnro 300/e9/06) and South Karelian Central Hospital (Dnro 06/08).

### Socioeconomic and health data

Study nurses in the maternity hospitals collected the data on marital status, education, smoking, alcohol consumption, and health, including information on chronic diseases (yes/no), with questionnaires. They also performed height and weight measurements at the follow-up visit and calculated the BMI for each patient based on these parameters. Women with a BMI ≥ 30 kg/m^2^ were defined as currently affected by obesity. If the woman had been recruited to the original RADIEL intervention because of previous GDM and/or been diagnosed with GDM during the RADIEL study, we defined her as GDM+.

### Physical activity

We monitored the amount of sleep, total steps/day, sedentary time, LPA, MVPA, and VPA with a SenseWear Armband™ PA monitor for seven consecutive days. The estimate parameters were extracted using proprietary algorithms (SenseWear professional software). The Metabolic Equivalent (MET) cutoff points were for sedentary time < 1.5 MET, LPA 1.5–3.0 MET, MVPA > 3.0 MET, and for VPA > 6.0 MET. Results were expressed as weighted averages.

The SenseWear Armband™ is a multisensor monitor worn on the upper arm over the triceps muscle. It continuously records data from four sensors on the armband (skin temperature, galvanic skin response, heat flux, and near-body temperature) as well as by accelerometery [22–24]. The monitor combines accelerometer data with other heat-related sensors to assess PA, sedentary time, and sleep. The Sense Wear softwear estimates energy expenditure (EE) from each minute of data using complex pattern-recognition algorithms. Previous study has demonstrated that the Sense Wear Armband™ provides valid estimates of EE, but less is known about its performance with specific intensities or types of movements [25]. The monitor provides information on how many hours data is measured per day, and how much of this data is with monitor on the body.

The results were considered valid and accepted for further analysis if they included measurements on at least three measured days during weekdays (WD) and one day during the weekend (WE). We defined as valid measurement days those that included > 12 h of measured data with at least 85% coverage. The total amount of daily PA (TotPA) was calculated as the weighted average of the PA of WD and WE ([5*WD + 2*WE]/7). The total average PA/week was calculated as 7*TotPA/day.

### Health-related quality of life

We used the 15D instrument to measure HRQoL. It is a generic instrument that can be used both as a profile and as a single index score measure and the Finnish version has been validated for adults (age 16 years +) [26]. The repeatability coefficients for 15D are 92–100% depending on dimensions [26]. 15D instrument has been used to measure health status and HRQoL of diverse patients, population groups, and populations [26], including individuals at risk for T2D [12, 13]. The 15D includes 15 HRQoL dimensions (mobility, vision, hearing, breathing, sleeping, eating, speech, excretion, usual activities, mental function, discomfort and symptoms, depression, distress, vitality, and sexual activity). The respondent chooses the value describing their current state of health related to each dimension on a scale of 1 (best) to 5 (worst). The valuation system of the 15D is based on multiattribute utility theory, and the single index 15D score on a scale of 0 (being dead) to 1 (full health) is calculated from the raw values using population-based preference or utility weights. A clinically important minimum change in the 15D score is +/− 0.015, representing the smallest difference that patients perceive as beneficial or harmful [27].

### Data analysis

We present the results as mean and standard deviation (SD) and median and interquartile range (IQR) when appropriate. The normality of the continuous variables was tested visually and with the Kolmogorov–Smirnov test. For the comparisons, we divided the sample into acknowledged T2D risk groups; first according to their current BMI (< 30 kg/m^2^ and ≥ 30 kg/m^2^) and second according to their GDM status (GDM+/GDM−). Finally, we made a separate group of women with both risk-factors (BMI ≥ 30 kg/m^2^ and GDM+). We conducted between-group comparisons using the Mann–Whitney U-test or the Chi-square test as appropriate. As the data on device-measured PA and 15D were non-normally distributed, we tested the crude associations with Spearman’s correlation and with Spearman’s partial correlation when adjusting for confounding variables (age, BMI when appropriate, number of children, chronic diseases (yes/no), smoking (yes/no), and total years of education (< 9 years, 9–12 years, 13–16 years, and > 16 years), and whether women were randomized to control or intervention group in the original study). Statistical significance was accepted at *p* < 0.05 (Bonferroni correction applied). Strength of correlation was determined poor if r_s_ < 0.3, fair if r_s_ = 0.3 to 0.5, moderately strong if r_s_ = 0.6 to 0.8, and strong if r_s_ = at least 0.8 [28]. All analyses were performed using IBM SPSS version 25.

## Results

### Participant characteristics

A total of 348 of the invited 596 women participated in the follow-up study; of these, 204 had both valid PA and 15D data available. We included these 204 women in the final analyses. When we compared the data of these enrolled subjects compared to the original RADIEL-study sample (N = 720), the mean age of enrolled subjects was higher in the original study (33.2 vs. 31.9, *p* < 0.001) and they were more educated (*p* = 0.001), but otherwise the groups were comparable*.*

The enrolled participants’ characteristics are presented in Table 1. The median age of the women was 39 (IQR 6) years, and the median BMI was 31.1 (IQR 10.9) kg/m^2^. Almost 47% percent (n = 96) of women in this study sample were randomized to the intervention group in the original study. Fifty-four percent of the women were currently affected by obesity. When we divided the women into two groups according to their BMI (< 30 kg/m^2^ or ≥ 30 kg/m^2^), the background characteristics between the BMI groups did not differ, except for number of children (*p* < 0.001), and chronic diseases requiring medication (*p* < 0.05).

**Table 1:** Characteristics of all women with obesity and/or prior gestational diabetes, by BMI and GDM groups, and in BMI ≥ 30 kg/m^2^ AND GDM+ -group

Character	Whole population (N = 204)	BMI < 30 kg m^−2^ (N = 94)	BMI ≥ 30 kg m^−2^ (N = 110)	*p* Value*	GDM− (N = 62)	GDM+ (N = 142)	*p* Value*	BMI ≥ 30 kg m^−2^ AND GDM+ (N = 58)	*p* Value*
Age, years, Median, (IQR)	39 (6.0)	39 (6.0)	38.5 (6.0)	0.89	38 (6.0)	39.0 (5.0)	0.08	40 (5)	0.086
BMI, kg m^−2^, median, (IQR)	31.1 (10.9)	25.0 (4.4)	35.8 (6.0)		35.0 (7.8)	28.7 (9.3)	** < 0.001**	34.5 (5.2)	** < 0.001**
15D score, median (IQR)	0.934 (0.092)	0.947 (0.103)	0.921 (0.083)	** < 0.001**	0.930 (0.095)	0.939 (0.090)	0.72	0.927 (0.086)	0.277
Intervention-group in the original study (%)	96 (47.1%)	49 (52.1%)	47 (42.7%)	0.16	27 (43.5%)	69 (48.6%)	0.61	22 (37.9%)	0.100
Years of education number (%)				0.18			0.34		0.553
Less than 9 years	3 (1.5%)	2 (2.1%)	1 (0.9%)		0 (0%)	3 (2.1%)		1 (1.7%)	
9–12 years	19 (9.3%)	7 (7.5%)	12 (10.9%)		5 (8.1%)	14 (9.9%)		8 (13.8%)	
13–16 years	56 (27.5%)	22 (23.4%)	34 (30.9%)		23 (37.1%)	33 (23.2%)		16 27.6%)	
More than 16 years	126 (61.8%)	63 (67.0%)	63 (57.3%)		34 (54.8%)	92 (64.8%)		33 (56.9%)	
Married/in a registered relationship/cohabiting (yes), number (%)	186 (91.1%)	89 (94.7%)	97 (88.2%)	0.10	57 (91.9%)	129 (90.8%)	0.06	49 (84.5%)	0.068
Median number of children (< 18y) in the family, mean (min–max, IQR)	2 (1–5, 1)	2 (1–5, 1)	2 (1–4, 0)	**0.032**	2 (1–4, 1)	2 (1–5, 1)	** < 0.001**	2 (1–4, 1)	0.539
Smoking regularly (yes), number (%)	11 (5.4%)	3 (3.2%)	8 (7.3%)	0.20	4 (6,5%)	7 (4.9%)	0.65	4 (6.9%)	0.549
Prior GDM (yes), number (%)	140 (68.6%)	82 (87.2%)	58 (52.7%)	** < 0.001**					
Reported one or more chronical disease (yes), number (%)	62 (30.4%)	20 (21.3%)	42 (38.2%)	**0.018**	23 (37.1%)	39 (27.5%)	0.17	27 (46.6%)	0.372
Asthma or COPD (chronic obstructive pulmonary disease)	15 (7.4%)	6 (6.4%)	9 (8.2%)		5 (8.1%)	10 (7.0%)		5 (8.6%)	
Migraine	14 (6.9%)	6 (6.4%)	8 (7.3%)		2 (3.2%)	12 (8.5%)		8 (13.8%)	
Depression	13 (6.3%)	3 (3.2%)	10 (9.1%)		5 (8.1%)	8 (5.6%)		5 (8.6%)	
Hypothyreoidism	12 (5.9%)	4 (4.3%)	8 (7.3%)		6 (9.7%)	6 (4.2%)		4 (6.9%)	
Hypertension	11 (5.4%)	1 (1%)	10 (9.1%)		4 (6.5%)	7 (4.9%)		6 (10.3%)	
Diabetes	5 (2.5%)	2 (2.1%)	3 (2.7%)		1 (1.6%)	4 (2.8%)		2 (3.4%)	
Arrythmias	3 (1.5%)	2 (2.1%)	1 (0.9%)		1 (1.6%)	2 (1.4%)		0 (0%)	
Rheumatoid arthritis	2 (1%)	1 (1%)	1 (0.9%)		1 (1.6%)	1 (0.7%)		0 (0%)	
Cancer	1 (0.5%)	1 (1.1%)	0 (0%)		0 (0%)	1 (0.7%)		0 (0%)	
Other	18 (8.8%)	9 (9.6%)	9 (8.2%)		6 (9.7%)	12 (8.5%)		7 (12.1%)	

*BMI* body mass index, *GDM* gestational diabetes, *IQR* interquartile range

*p Value for the difference between the BMI-groups and GDM-groups, and between BMI ≥ 30 kg/m^2^ and GDM+ group and whole sample, Mann–Whitney U test or Chi Square test

Significant *p* Values were bolded

Of all the women, 70% had a confirmed diagnosis of prior GDM, and, of these, 29% were also currently affected by obesity (BMI ≥ 30 kg/m^2^). The GDM+ women, when compared to the GDM− women, were on average leaner (median BMI 28.7 kg/m^2^ vs. 35.0 kg/m^2^, *p* < 0.001) and had more children in the household (mean 1.87 vs. 2.47 children, *p* < 0.001) (Table 1). We also present the background characters of those women that had both risk-factors (current BMI ≥ 30 kg/m^2^ and GDM+) in Table 1.

### Physical activity

Table 2 shows the results of the armband measurements according to the BMI groups (BMI < 30 kg/m^2^ and BMI ≥ 30 kg/m^2^), the GDM- and GDM+ groups, and in the group of GDM+ women with current obesity. On average, during valid measuring days, the monitor was worn for 22.8 hours/day (19.8–24.0, SD 1 h/day). The median daily minutes of PA at all intensity levels (LPA, MVPA, and VPA) and the sleep time were lower in women with obesity (BMI ≥ 30 kg/m^2^) compared to women with normal weight to overweight (BMI < 30 kg/m^2^) (*p* < 0.001). In addition, the median sedentary time in the obese group was higher than in the normal weight to overweight group. Women with both obesity and GDM+ had significantly higher sedentary time and lower PA levels (LPA, MVPA, and VPA), compared to women with only either BMI ≥ 30 kg/m^2^ or GDM+ (Table 2).

**Table 2:** Armband outcomes in all women with obesity and/or prior GDM groups, and in BMI ≥ 30 kg/m^2^ and GDM group

ArmBand-outcomes	Whole sample (N = 204) Median (min–max; IQR)	BMI < 30 kg m^−2^ (N = 94) Median (IQR)	BMI ≥ 30 kg m^−2^ (N = 110) Median (IQR)	*p* Value^a^	GDM− (N = 62) Median (IQR)	GDM+ (N = 142) Median (IQR)	*p* Value^a^	BMI ≥ 30 kg m^−2^ AND GDM+ (N = 59) Median (IQR)	*p* Value^a^ (compared to whole sample)
Sleep, hours/day	6.8 (2.7–10.1; 1.1)	7.0 (1.2)	6.6 (1.2)	0.036*	6.6 (1.3)	6.8 (1.1)	0.280	6.5 (1.1)	0.008*
Total steps/day	8582 (3233–21,769; 4265)	9358 (4432)	8111 (3584)	0.034*	8787 (3057)	8539 (4379)	0.440	7856 (3516)	0.014*
Sedentary time (sleep not included), min/day	619.3 (324.2–989.5; 175.4)	553.0 (139.0)	673.4 (164.2)	< 0.001**	620.6 (162.3)	615.0 (175.9)	0.877	695.8 (172.6)	< 0.001**
LPA, min/day	258.0 (87.6–490.0; 120.7)	282.0 (91.0)	237.9 (132.0)	< 0.001**	262.9 (109.0)	257.7 (129.1)	0.877	224.5 (146.4)	0.012*
MVPA, min/day	64.9 (1.6–383.0; 58.5)	91.0 (57.0)	44.3 (33.4)	< 0.001**	56.4 (35.8)	70.5 (69)	0.042*	41.8 (37.0)	< 0.001**
VPA, min/day	0.3 (0–51.9; 2.8)	1.8 (8.0)	0.0 (0.5)	< 0.001**	0.2 (1.5)	0.4 (3.7)	0.371	0.00 (0.4)	< 0.001**

*GDM* gestational diabetes, *BMI* body mass index, *PA* physical activity, *IQR* interquartile range, *LPA* light physical activity, *MVPA* moderate-to-vigorous physical activity, *VPA* vigorous physical activity

GDM+  = GDM in prior pregnancy

GDM−  = No prior GDM

^a^*p* Value from Mann–Whitney U test. Difference between the BMI groups, the GDM-groups and between BMI ≥ 30 kg/m^2^ and GDM+ group and the whole sample

*p Value < 0.05

**p Value < 0.001

When we compared the PA levels between the GDM+ and the GDM- women, the median PA was different only for MVPA, which was 80.9 (IQR 63.3) minutes/day in the GDM+ group and 50.8 min/day (IQR 39.9) in the GDM- group (*p* < 0.05). After adjusting this result for confounders (age, BMI, number of children, chronic diseases, education years, smoking, and control/intervention in the original study), the difference was not statistically significant. Randomization to the initial RADIEL intervention or control group was not associated with any PA variables in our study sample.

Considering the relationship between BMI and the armband measurements in the whole study sample, the crude results showed a poor negative linear trend for higher BMI and total sleep (r_s_ =  − 0.172, *p* < 0.05), total steps (r_s_ =  − 0.176, *p* < 0.05), and a fair to moderately strong negative linear trend for BMI and LPA (r_s_ =  − 0.298, *p* < 0.001), MVPA (r_s_ =  − 0.637, *p* < 0.001), and VPA (r_s_ =  − 0.536, *p* < 0.001), and a moderately strong positive linear trend for BMI and sedentary time (r_s_ = 0.564, *p* < 0.001).

### Health-related quality of life

The whole sample’s median 15D score was 0.934 (range 0.624–1.00, IQR 0.092). The median 15D score in the BMI < 30 kg/m^2^ group was 0.947 (IQR 0.103) and 0.921 (IQR 0.083) in the BMI ≥ 30 kg/m^2^ group (*p* < 0.001). Prior GDM status (+ / −) was not associated with total 15D score. In the BMI ≥ 30 kg/m^2^ and GDM+ group, the mean 15D score was 0.927 (IQR 0.086) (Table 1). Also, randomization to the intervention or control group of the initial RADIEL intervention was not associated with current 15D score in our study sample. In the whole study sample, there was a poor negative linear correlation between BMI and total 15D score (r_s_ =  − 0.231, *p* < 0.001).

**Fig. 1:**
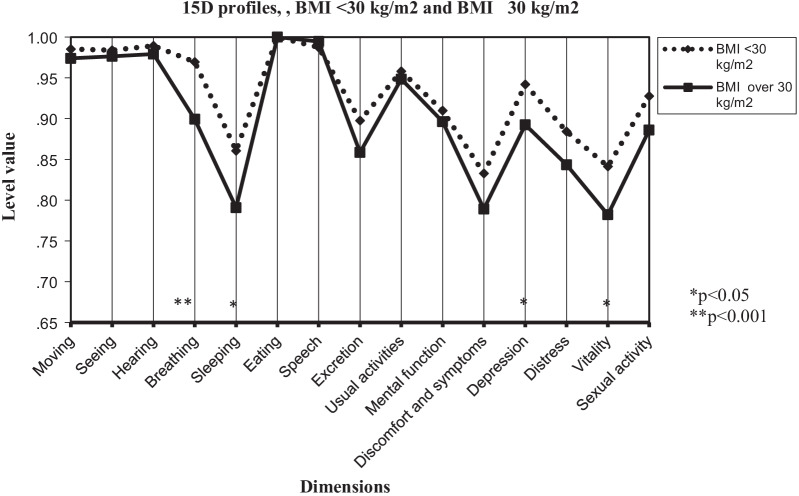
15D profiles of women with BMI < 30 kg/m^2^ and BMI ≥ 30 kg/m^2^; scores presented as means

**Fig. 2:**
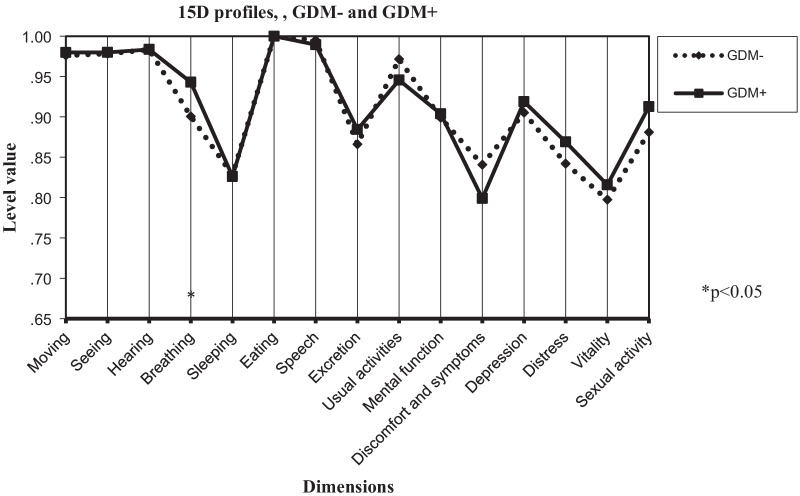
15D profiles of GDM- and GDM+ women; scores presented as means

Figure 1 shows the 15D dimension profiles in the BMI groups (< 30 kg/m^2^ and ≥ 30 kg/m^2^), and Fig. 2 shows the GDM- and GDM+ groups*.* Differences in the dimensions of breathing (*p* < 0.001), sleeping (*p* < 0.05), depression (*p* < 0.05), and vitality (*p* < 0.05) account for the differences in the total HRQoL scores between the BMI groups. Only the breathing dimension (*p* < 0.05) differed between the GDM+ and GDM− groups.

### Association between physical activity and health-related quality of life (15D score)

We present the correlations of the armband measurements of PA and the 15D score in Table 3. In the whole sample, there were positive linear correlations between the total number of daily steps (r_s_ = 0.200, *p* < 0.05), MVPA (r_s_ = 0.201, *p* < 0.05), VPA (r_s_ = 0.313, *p* < 0.001), and the 15D score.

**Table 3:** Associations between armband outcomes and 15D score in all women with obesity and/or prior GDM; by BMI groups, GDM groups, and in BMI > 30 kg/m^2^ and GDM+ group

	Crude^a^						Adjusted^b^	Adjusted^c^		Adjusted^b^		Adjusted^c^
Armband outcomes	All women	BMI < 30 kg m^−2^	BMI ≥ 30 kg m^−2^	GDM+	GDM−	BMI ≥ 30 kg m^−2^ and GDM+	All women	BMI < 30 kg m^−2^	BMI ≥ 30 kg m^−2^	GDM+	GDM−	BMI ≥ 30 kg m^−2^ and GDM+
Sleep	− 0.082 *p* = 0.241	− 0.255 *p* = 0.013*	− 0.026 *p* = 0.790	− 0.187 *p* = 0.027*	0.171 *p* = 0.192	− 0.255 *p* = 0.054	− 0.085 *p* = 0.243	− 0.246 *p* = 0.046*	0.043 *p* = 0.673	− 0.217 *p* = 0.028*	0.151 *p* = 0.279	− 0.165 *p* = 0.257
Total steps/day	0.200 *p* = 0.004*	0.317 *p* = 0.002*	0.018 *p* = 0.850	0.224 *p* = 0.008*	0.170 *p* = 0.195	0.031 *p* = 0.819	0.203 *p* = 0.050*	**0.361** *p* = 0.001**	− 0.031 *p* = 0.764	0.229 *p* = 0.09	0.203 *p* = 0.145	0.106 *p* = 0.470
Sedentary time	− 0.080 *p* = 0.253	− 0.042 *p* = 0.690	0.119 *p* = 0.217	− 0.04 *p* = 0.961	− 0.260 *p* = 0.045	0.298 *p* = 0.023*	0.014 *p* = 0.853	− 0.126 *p* = 0.250	0.103 *p* = 0.312	− 0.084 *p* = 0.345	− 0.250 *p* = 0.071	0.053 *p* = 0.053
LPA	0.068 *p* = 0.335	0.048 *p* = 0.649	− 0.040 *p* = 0.681	− 0.016 *p* = 0.848	0.274 *p* = 0.034*	− 0.172 *p* = 0.197	0.041 *p* = 0.577	0.122 *p* = 0.265	− 0.057 *p* = 0.577	− 0.028 *p* = 0.751	0.232 *p* = 0.094	− 0.225 *p* = 0.120
MVPA	0.201 *p* = 0.004*	0.139 *p* = 0.181	0.036p = 0.712	0.215 *p* = 0.011*	0.156 *p* = 0.234	0.059 *p* = 0.662	0.097 *p* = 0.194	0.212 *p* = 0.051	0.016 *p* = 0.874	0.129 *p* = 0.148	0.136 *p* = 0.330	0.095 *p* = 0.515
VPA	0.313 *p* < 0.001**	0.380 *p* < 0.001**	0.043 *p* = 0.658	0.330 *p* = 0.000**	0.285 *p* = 0.027*	− 0.064 *p* = 0.632	0.262 *p* < 0.001**	0.426 *p* < 0.001**	0.044 *p* = 0.666	**0.310** *p* = 0.001**	0.200 *p* = 0.150	− 0.011 *p* = 0.942

*LPA* light physical activity, *MVPA* moderate-to-vigorous physical activity, *VPA* vigorous physical activity

^a^Spearman's correlations

^b^Spearman’s partial correlations after adjustment for confounders: body mass index, age (years), number of children, smoking (yes/no), education (less than 9 years, 9–12 years, 13–16 years, and > 16 years), and chronic disease (yes/no), intervention/control group in the original study

^c^Spearman’s partial correlations after adjustment for confounders: age (years), number of children, smoking (yes/no), education (less than 9 years, 9–12 years, 13–16 years, and > 16 years), and chronic disease (yes/no), intervention/control group in the original study

Significant adjusted results were bolded

*p < 0.05

**p < 0.001

After we adjusted the results for confounders (age, BMI, number of children, chronic diseases, education years, smoking, and intervention/control -group in the original study), the positive correlations between VPA (r_s_ = 0.262, *p* < 0.001) and total steps/day (r_s_ = 0.203, *p* = 0.05) and the 15D score remained significant in the whole sample (Table 3).

When we adjusted the results from the two BMI groups (< 30 kg/m^2^ or ≥ 30 kg/m^2^) for the confounders (age, number of children, chronic diseases, education years, smoking and intervention/control group in the original study), the correlations between PA and the 15D score were significant only for the group with a BMI < 30 kg/m^2^. Among them, sleep correlated fairly (r_s_ =  − 0.246, *p* < 0.05), and total steps (r_s_ = 0.361, *p* < 0.001), and VPA moderately strongly (r_s_ = 0.426, *p* < 0.001) with the 15D score. In addition, in the GDM+ group, the fair positive correlation between VPA (r_s_ = 0.310, *p* = 0.001) and the poor negative correlation between total sleeping time (r_s_ =  − 0.217, *p* < 0.05) and the 15D score remained after adjusting for the confounders (BMI, age, number of children, chronic diseases, education years, smoking, control/intervention group in the original study) (Table 3). In the group of women with both obesity and GDM+, there were no significant correlations between armband measurements and 15D score after the adjustments (Table 3).

### Association between physical activity and 15D dimensions

In the whole sample, in the unadjusted results, the 15D dimension of vitality correlated poorly with MVPA (r_s_ = 0.216, *p* < 0.05) and fairly with VPA (r_s_ = 0.374, *p* < 0.001). We also detected negative correlations between total sleep time and the dimensions of usual activities (r_s_ =  − 0.269, *p* < 0.05), vitality (r_s_ =  − 0.249, *p* < 0.05), and breathing (r_s_ =  − 0.234, *p* < 0.05).

As BMI, but not GDM, was associated with HRQoL in the whole sample, and PA correlated with 15D score in the BMI < 30 kg/m^2^ group, we examined the associations between PA and 15D dimensions in the BMI < 30 group. The adjusted results for the BMI < 30 kg/m^2^ group showed positive correlations especially between total steps and VPA and 15D dimension (Table 4). In particular, positive correlations between VPA and dimension of vitality (r_s_ = 0.492, *p* < 0.001) and total steps (r_s_ = 0.374, *p* < 0.001) were detected. VPA also correlated fairly with decreased symptoms and discomfort (r_s_ = 0.447, *p* < 0.001), and distress (r_s_ = 0.310, *p* < 0.05). Also, there were poor positive correlations between VPA and the dimensions of excretion (r_s_ = 0.281, *p* < 0.05), and breathing (r_s_ = 0.264, *p* < 0.05). In addition to the dimension of vitality, total steps correlated with better health status on the dimensions of distress (r_s_ = 0.384, *p* < 0.001), discomfort and symptoms (r_s_ = 0.291, *p* < 0.05), and excretion (r_s_ = 0.247, *p* < 0.05).

**Table 4:** Spearman’s correlations between armband outcomes and 15D dimensions in women with obesity and/or prior GDM

Armband	Mobility	Breathing	Usual activities	Excretion	Discomfort and symptoms	Sex	Distress	Vitality
Total steps/day	0.163 (*p* = 0.116)	0.181 (*p* = 0.081)	0.092 (*p* = 0.376)	0.281 (*p* = 0.006) **0.247**^**a**^ **(*p* = 0.046)**	0.233 (*p* < 0.024) **0.291**^**a**^ **(*p* = 0.014)**	0.127 (*p* = 0.224)	0.281 (*p* = 0.006) **0.384**^**a**^ **(*p* = 0.000)**	0.347 (*p* < 0.001) **0.374**^**a**^ **(*p* = 0.000)**
Sleep	− 0.168 (*p* = 0.105)	0.252 (*p* = 0.014) − 0.228^a^ (*p* = 0.072)	0.239 (*p* = 0.023) ** − 0.251**^**a**^ **(*p* = 0.042)**	− 0.166 (*p* = 0.018) − 0.123 (*p* = 0.263)	0.032 (*p* = 0.763)	− 0.113 (*p* = 0.280)	− 0.160 (*p* = 0.124)	− 0.279 (*p* = 0.006) ** − 0.261**^**a**^ **(*p* = 0.032)**
Sedentary time	− 0.69 (*p* = 0.510)	− 0.52 (*p* = 0.622)	0.013 (*p* = 0.901)	− 0.155 (*p* = 0.135)	0.01 (*p* = 0.990)	0.001 (*p* = 0.996)	− 0.710 (*p* = 0.499)	− 0.143 (*p* = 0.169)
LPA	0.141 (*p* = 0.176)	0.054 (*p* = 0.606)	0.104 (*p* = 0.317)	0.079 (*p* = 0.450)	0.060 (*p* = 0.957)	− 0.034 (*p* = 0.748)	− 0.010 (*p* = 0.926)	0.079 (*p* = 0.497)
MVPA	0.78 (*p* = 0.454)	0.100 (*p* = 0.335)	0.240 (*p* = 0.821)	0.187 (*p* = 0.072)	0.029 (*p* = 0.781)	0.030 (*p* = 0.771)	0.106 (*p* = 0.311)	0.248 (*p* = 0.0016) **0.262**^**a**^ **(*p* = 0.026)**
VPA	0.207 (*p* = 0.051)	0.261 (*p* < 0.011) **0.264**^**a**^ **(*p* = 0.028)**	0.108 (*p* = 0.299)	0.222 (*p* = 0.032) **0.281**^**a**^ **(*p* = 0.018)**	0.350 (*p* = 0.001) **0.447**^**a**^ **(*p* = 0.000)**	0.209 (*p* = 0.043) 0.219^a^ (*p* = 0.088)	0.142 (*p* = 0.043) **0.310**^**a**^ **(*p* = 0.008)**	0.4976 (*p* < 0.000) **0.492**^**a**^ **(*p* < 0.000)**

Armband	Vision	Hearing	Sleeping	Eating	Speech	Mental function	Depression
Total steps/day	0.158 (*p* = 0.129)	0.008 (*p* = 0.941)	0.151 (*p* = 0.146)	0.140 (*p* = 0.137)	0.113 (*p* = 0.280)	0.069 (*p* = 0.508)	0.109 (*p* = 0.297)
Sleep	− 0.117 (*p* = 0.260)	0.080 (*p* = 0.941)	0.149 (*p* = 0.151)	0.167 (*p* = 0.278)	0.074 (*p* = 0.480)	− 0.171 (*p* = 0.99)	0.184 (*p* = 0.076)
Sedentary time	− 0.019 (*p* = 0.858)	0.103 (*p* = 0.323)	0.075 (*p* = 0.472)	0.182 (*p* = 0.350)	− 0.031 (*p* = 0.766)	0.082 (*p* = 0.433)	0.025 (*p* = 0.810)
LPA	0.095 (*p* = 0.363)	0.051 (*p* = 0.629)	0.024 (*p* = 0.819)	0.122 (*p* = 0.285)	− 0.105 (*p* = 0.314)	0.072 (*p* = 0.491)	0.086 (*p* = 0.408)
MVPA	0.107 (*p* = 0.306)	0.019 (*p* = 0.853)	0.064 (*p* = 0.537)	0.002 (*p* = 0.953)	0.109 (*p* = 0.297)	− 0.050 (*p* = 0.629)	0.106 (*p* = 0.311)
VPA	0.180 (*p* = 0.083)	− 0.006 (*p* = 0.955)	0.192 (*p* = 0.064)	− 0.091 (*p* = 0.384)	0.135 (*p* = 0.193)	0.072 (*p* = 0.489)	0.247 (*p* = 0.016) 0.235^a^ (*p* = 0.062)

Women with BMI < 30 kg/m^2^, unadjusted and adjusted results

*LPA* light physical activity, *MVPA* moderate-to-vigorous physical activity, *VPA* vigorous physical activity

Significant adjusted results were bolded

^a^Spearman’s partial correlations after adjustment for confounders; age (years), smoking (yes/no), number of children, education (less than 9 years, 9–12 years, 13–16 years, and > 16 years), chronic disease (yes/no), and intervention/control group in the original study

## Discussion

To the best of our knowledge, this is the first study to evaluate the association between measured PA and HRQoL in high-risk (prior GDM and/or current obesity) women during the early years after pregnancy when their risk of T2D is highest. We showed that obesity was negatively associated with PA levels and HRQoL. Prior GDM status was not associated with PA or total HRQoL score in our study. Women with both prior GDM and obesity had higher sedentary time and lower PA levels compared to other women. PA was positively associated with total HRQoL scores in the women with normal weight and overweight but not in the women with obesity. VPA was also positively associated with total HRQoL score among women with prior GDM, but not among women with both prior GDM and obesity. We detected fair correlations between VPA and the dimensions of vitality, discomfort and symptoms, and distress among the women with normal weight or overweight. In addition, total steps correlated moderately with the vitality dimension in the women with normal or overweight.

A negative correlation between BMI and HRQoL has also been found in previous studies [12, 19, 20], although those studies included older subjects from both genders. Evidence suggests that the impact of obesity on HRQoL and its components is related to age and gender [19]. In our sample, the mean 15D score was lower than in age-matched Finnish women in a prior population-based study (N = 5800) and similar to that of women aged 55 years or older [29]. In that previous study [29], the mean BMI of the age-matched respondents was, however, lower (25.4 kg/m^2^) than in our study, which can explain the difference, as women with obesity and overweight seem to be especially prone to depressed mood and anxiety [30] and deterioration of HRQoL [19, 20].

In agreement with the results of the study by Halkoaho et al. [31], which examined women approximately 64–66 months postpartum, prior GDM status was not associated with general HRQoL in our sample either. Also, similar to our study, GDM status during pregnancy has not been associated with women’s HRQoL [32]. This observation seems logical, as GDM or early T2D are not usually associated with significant symptoms [1, 2]. Compared to the study by Väätäinen et al., which reported an association between T2D risk and HRQoL [13], our sample included only younger women, which in turn may be reflected in the differences in PA, comorbidities, and general HRQoL between the samples [8, 11].

The positive association between PA and HRQoL found in our study is in line with previous literature concerning general populations [8, 9, 11], older populations at risk for T2D [12], and women planning pregnancy and at risk for GDM [33]. The adjusted association between PA and HRQoL was, however, only seen in women with normal weight and overweight in our study, but not in women with obesity. In contrast, in a Canadian cross-sectional survey [11], PA correlated positively with HRQoL, regardless of weight status. Furthermore, lower levels of PA have been associated with poorer HRQoL among inactive women among the general population [34], and meeting the recommended PA level has been associated with better HRQoL (evaluated with the Behavioral Risk Factor Surveillance System, BRFSS) in the overweight and obese population [35]. In contrast to our study, these previous studies used self-reported questionnaires instead of device measurement when determining PA.

Daily PA levels in the current study were higher than those reported in previous population-based Finnish cohort studies of the same gender and age group [36, 37] that used different methods: monitoring daily steps with a step monitor [36] and the amount of MVPA with a hip-worn accelerometer [37]. Even though with a higher BMI, the amount of PA tended to decrease, the amount of measured MVPA seemed to be relatively high in our study population. In our study, GDM+ women were leaner than GDM- women, and the level of education in our sample was somewhat high. This may partly explain their relatively high PA and 15D score levels as, in addition to BMI, better socioeconomic status, even in high-income countries, enables people to make healthier lifestyle choices, leading to social gradients of health [18, 38–40]. PA levels among high-risk women in our study were higher compared to women with recent GDM in a study by Smith et al. [17]. However, they assessed PA with a questionnaire and at 6–24 months postpartum compared to using a multisensory monitor 4–6 years postpartum in our study. Having longer time from the delivery and different PA assessment methods may explain the higher PA levels detected in our study. The earlier studies all used different methods to measure PA, and therefore the results are not directly comparable with our results.

The difference in total HRQoL scores between the BMI groups in our study was explained mainly by differences in the dimensions of vitality, sleeping, depression, and breathing. These observations are also in agreement with previous studies [19, 20], even though different HRQoL instruments may be sensitive to different health dimensions [41].

Considering the associations between PA and the dimensions of HRQoL found in our study, similar associations between PA and vitality have also been detected in previous studies in the general population [42], a population with metabolic risk factors [43], and T2D patients [44]. We speculate that this positive association demonstrates the potential bi-directional positive associations between PA and the sense of vitality; when an individual perceives herself as being vital, she is more likely to engage in PA, which in turn can increase her sense of vitality. These potential bi-directional associations can also explain our results concerning the correlations between PA and distress, discomfort and symptoms as previous community-based study has shown that low PA levels are associated with higher prevalence of anxiety [45], and on the other hand PA has been shown to decrease anxiety and stress symptoms [46]. The positive association between VPA and breathing could, in turn, be explained merely by better physical health and cardiorespiratory fitness caused by PA. Also, overweight is known to impair respiratory health in mechanical and inflammatory ways [47]. The positive associations concerning PA and different dimensions of HRQoL are also in line with the study by Häkkinen et al. [12], which detected benefits from self-reported PA for all HRQoL dimensions (assessed with the SF-36) among older people with elevated T2D risk [12].

According to previous research, women affected by obesity may be more prone to a depressed mood [30, 48]. In our study, the 15D depression dimension showed a significant difference between the BMI groups. Even though the difference in the whole sample did not remain significant after adjusting for confounders, VPA correlated in our crude results with less depressive symptoms. Depression results in deterioration of HRQoL [49], and epidemiological data suggest an association between obesity and depression [50]. People with depression also tend to live more sedentary lives [51], and PA has been reported to diminish depressive symptoms [51]. The possible causal interactions between these factors are multidirectional, complicated, and impossible to distinguish in our study, however.

In addition to lower PA levels, the women affected by obesity in our study had shorter sleep during the night than the leaner group, which may have reflected directly on the HRQoL dimension of sleep. As PA has positive effects on total sleep time and quality [52], we think that it is not surprising that daily PA and sedentary time were also reflected in the sleep dimension in our study. An optimal amount of sleep has been shown to reflect positively on general HRQoL [53].

Strengths of our study were that PA was device-measured and the women’s heights and weights were measured, in contrast to many previous studies using self-reported questionnaires. Also, our sample represents high-risk women during the years of high risk for T2D. This period for these women is a true window of opportunity for healthy lifestyle choices, choices that can concretely reduce their risk of developing T2D.

The limitations of our study include the fact that the women in our study volunteered and may represent a more physically active group of women than the general high-risk population. Furthermore, it is possible that women changed their PA habits during the measurement period. Also, as the high-risk women affected by obesity spent significantly less time in MVPA and VPA than the high-risk women with normal weight or overweight, it is possible that the amount of higher-intensity PA was simply not sufficient to be reflected in the 15D scores. The lack of a priori sample size calculations for these particular outcomes in this follow-up study can also be considered as a limitation. Also, we cannot rule out the possibility of interactions between variables. Finally, when estimating HRQoL, different instruments exhibit varying sensitivity to different dimensions of HRQoL, which may be reflected in the results. Conversely, 15D has previously been used in people with different conditions and Finnish population-based studies and is comparable to other preference-based generic HRQoL instruments [26].

## Conclusions

In conclusion, in our sample of premenopausal women at high risk for T2D, current obesity, but not prior GDM status, correlated with HRQoL four to six years after pregnancy. Higher PA levels were associated with better HRQoL in women with normal weight and overweight, suggesting that a more physically active lifestyle is positively associated with subjective well-being. The associations between PA and HRQoL were, however, not seen in women with obesity who engaged in low levels of VPA. When considering the correlation between PA and HRQoL, our results suggest that BMI, instead of history of GDM is more significant factor.

Women in our sample had previously volunteered to receive individualized counselling on healthy life-style choices during their pregnancy and were currently compliant to the follow-up. It would be reasonable to speculate that these women are competent and willing to view and review their lifestyle choices. Even though, increasing BMI correlated negatively with PA and especially those women with both current obesity and prior GDM were more sedentary than other high-risk women.

Future studies should investigate how to reach and motivate these high-risk women into long-term life-style changes. These high-risk women could especially benefit from feasible, individualized, and motivating interventions aimed at increasing commitment to MVPA and VPA during this very special period of early motherhood. This could contribute to decreasing their future risk of developing T2D and simultaneously modify women´s subjective well-being and HRQoL. In addition to individual impact, these interventions may translate into valuable societal benefits by reducing the unequal morbidity associated with physical inactivity and obesity.

## Data Availability

The datasets generated and/or analyzed during the current study are not publicly available due to the lack of informed consent of the participants to release the data, but they are available from the corresponding author on reasonable request.

## Abbreviations

BMI: Body mass index
EE: Energy expenditure
GDM: Gestational diabetes
HRQoL: Health related Quality of Life
LPA: Light physical activity
MVPA: Moderate-to-vigorous physical activity
PA: Physical activity
T2D: Type 2 diabetes
VPA: Vigorous physical activity
